# Neuroform Atlas stent-assisted coiling of ruptured wide-necked anterior communicating artery aneurysms

**DOI:** 10.3389/fneur.2025.1674012

**Published:** 2025-10-08

**Authors:** Hongping Wang, Shengli Hu, Jianyi Xu, Jian Pei, Yunhe Gao, Baogang Tian, Dayong Wang, Yu Zheng, Xiang Xu

**Affiliations:** ^1^Department of Neurosurgery, Tangshan Workers’ Hospital, Tangshan, Hebei, China; ^2^Department of Neurosurgery, Taihe Hospital Affiliated to Hubei University of Medicine, Shiyan, Hubei, China; ^3^Department of Biostatistics, University of Michigan, Ann Arbor, MI, United States

**Keywords:** ruptured intracranial aneurysm, wide-necked, anterior communicating artery, stent-assisted coiling, endovascular treatment, Neuroform Atlas stent

## Abstract

One of the most crucial methods for treating wide-neck anterior communicating artery aneurysms is endovascular therapy. However, because of the small arterial diameter and volume with hematoma, endovascular embolization procedures for the treatment of burst wide-necked ACoA aneurysms are difficult. Few studies have been conducted to evaluate the Neuroform Atlas stent’s safety and effectiveness in treating wide-necked anterior communicating artery aneurysms, despite the fact that it has been frequently utilized to treat wide-necked aneurysms since its 2020 debut in China. Thus, this article’s goal is to examine the effectiveness and safety of Neuroform Atlas stent-assisted coiling in the management of anterior communicating artery burst wide-necked aneurysms. From August 2020 to June 2022, Neuroform Atlas stents were used to treat 30 patients with aneurysms of the ruptured wide-necked anterior connecting arteries. Neuroform Atlas stents were successfully and satisfactorily positioned in each of the 30 patients. Three patients had postoperative complications. Twenty-nine patients (96.7%) achieved an mRS score of ≤2, demonstrating satisfactory clinical outcomes, while one patient (3.3%) had an mRS score of 3, indicating poor prognosis. This pilot research shows that coiling ruptured wide-necked aneurysms of the anterior communicating artery with the use of a Neuroform Atlas stent is safe and effective.

## Introduction

Anterior communicating artery (ACoA) aneurysms are the most common intracranial aneurysms and ACoA is one of the most common sites for rupture in intracranial aneurysms (1–3). A wide-neck aneurysm refers to an aneurysm with a neck width exceeding 4 millimeters or a body-to-neck ratio of less than 2:1 (4). Endovascular treatment has become an important method for managing these lesions (5–7). Compared with simple coil embolization, stent-assisted coiling (SAC) therapy helps achieve the safety of the procedure and promote healing of the aneurysm neck, thus reducing the risk of recurrence after surgery, especially for wide-necked aneurysms and aneurysms with complex shapes (8). However, due to the small vessel diameter, minimal volume, and associated hematoma, endovascular embolization techniques for ruptured wide-neck ACoA aneurysms remain challenging (9). Since the Neuroform Atlas stent (Stryker Neurovascular, Fremont, California) was launched in China in 2020, it has been widely used to treat wide-neck aneurysms. The Atlas stent is a self-expanding laser-cut nitinol stent delivered through a low-profile microcatheter (size, 0.0165–0.017 inches). Its novel mixed open-cell/closed-cell design enhances stability within the vessel, provides high flexibility, and ensures proper adhesion to the vessel wall (10).

Despite previous studies confirming the safety and efficacy of the Atlas stent (11, 12), few studies have focused on ruptured wide-neck aneurysms in the anterior communicating artery. This study aims to evaluate the safety and efficacy of using the Atlas stent-assisted embolization for treating ruptured wide-neck aneurysms in the anterior communicating artery, and to assess risk factors associated with procedure-related complications.

## Materials and methods

### Patient selection

We performed a retrospective analysis of 30 patients who underwent Atlas SAC of ruptured wide-necked ACoA aneurysms between August 2020 and June 2022 in the Department of Neurosurgery of Tangshan Workers’ Hospital Affiliated to Hebei Medical University. The study was approved by the ethics committee at the Tangshan Workers’ Hospital Affiliated to Hebei Medical University, China. The requirements for inclusion were as follows: (1) having a wide neck (neck diameter >4 mm or aneurysm dome to neck ratio <2) anterior communicating artery aneurysms verified by digital subtraction angiography; (2) having spontaneous subarachnoid hemorrhage (SAH) accompanied by abrupt nausea, vomiting, and severe headache; (3) having a cranial CT examination to confirm SAH; (4) having full case data.

DSA was used to identify all aneurysms, and a dome-to-neck ratio of less than two was considered wide-necked. Aneurysm form, position, rupture status, and dome-to-neck ratio were recorded by analyzing imaging data.

### Endovascular treatment

In order to do stent-assisted aneurysm embolization, all patients were put in a supine posture and given general anesthesia and systemic heparinization by a puncture of the right femoral artery. By evaluating the size, shape, and diameter of the aneurysm neck as well as the diameter of the parent artery, 3D rotational angiography was carried out intraoperatively to identify the best working path. In our current study describes two different techniques for stent placement (single and double microcatheter methods). Seven patients received treatment with a single microcatheter, while 23 patients received treatment with a double microcatheter. The chosen A2 section of the anterior cerebral artery was fitted with an SL-10 microcatheter (Stryker Cork, Ireland) Herniation prevents the aneurysms from being completely obliterated, and a single microcatheter may lead the coils to enter the parent artery for wide-necked aneurysms. Double-microcatheter coiling (DMC) is utilized in conjunction with incomplete occlusion to prevent coil compaction or herniation (13). In 25 cases of wide-neck aneurysms, the dual-microcatheter approach proved to be both feasible and safe for coil embolization of aneurysms with unfavorable topologies (14). The double microcatheter method Operational protocol: Insert a Neuroform Atlas stent (Stryker Neurovascular Cork, Ireland) using the SL-10 microcatheter, ensuring precise placement with the distal tip of the stent lands on the A2 segment. Next, shape the Echelon 10 microcatheter (Micro Therapeutics Inc. dba ev3 Neurovascular Company, United States) and position it inside the anterior communicating artery aneurysm sac. To cover the aneurysm’s neck, gradually remove the proximal end of the stent. Keep releasing until the aneurysm is fully separated. Once confirmed that the stent is fully expanded and covered the aneurysm’s neck, withdraw the SL-10 microcatheter. Coils are then constantly delivered through the Echelon 10 microcatheter to completely pack the aneurysm. For stent-assisted coiling (SAC) of wide-neck intracranial aneurysms with small-caliber, stenosis, or a particularly convoluted course in the parent artery, putting a single microcatheter and Neuroform Atlas stent within a 5 Fr (or smaller) guidewire or intermediate catheter may be an efficient method (15). The single microcatheter method Operation protocol: The Neuroform Atlas stent is delivered via the SL-10 microcatheter. The SL-10 microcatheter enters the aneurysm sac by passing through the mesh holes of the stent after it has fully inflated and covered the aneurysm’s neck. Coils are then constantly released by the microcatheter to achieve complete aneurysm filling. A total of 30 Neuroform Atlas stents were used to treat 30 patients with aneurysms, including 13 stents measuring 3 mm × 21 mm, 15 stents measuring 3 mm × 15 mm, and 2 stents measuring 4 mm × 15 mm. Immediate post-embolization angiography was performed for all patients, and the aneurysms’ embolization and the stents’ patency were assessed after a 10-minute interval.

### Clinical and radiographic outcomes

The Raymond-Roy (RR) occlusion grading method was used to assess the aneurysm’s embolization right away: Grade I denoted complete occlusion, Grade II denoted residual neck, and Grade III denoted residual aneurysm (16). The modified Rankin scale (mRS) was used to assess neurological recovery following surgery. A favorable outcome was defined as an mRS score ≤2, while a poor outcome was defined as a score ≥3. Thrombotic problems were described as delayed blood flow filling, in-stent filling defects, or non-visualization of the parent artery’s distal or branch arteries during the surgery. Extravascular leakage of contrast medium observed on intraoperative DSA was considered a hemorrhagic complication. Following the surgery, all patients underwent comprehensive neurological examination and head CT. Clinical follow-up was conducted either via modified Rankin Scale (mRS) interviews or visits to the neurosurgery outpatient clinic, with an mRS score ≤2 indicating a favorable functional outcome.

### Statistical analysis

Statistical analyses were performed using SPSS software version 26.0 (IBM Corp., Armonk, NY, United States). Continuous variables with a normal distribution are expressed as means with standard deviation (SD). Categorical variables are expressed as numbers with percentages. Continuous variables and rank order variables were compared using the Student’s *t*-test or Wilcoxon rank-sum test, as appropriate. Categorical variables were compared using the chi-square or Fisher’s exact test as appropriate. Variables with *p* < 0.1 were included in the multivariate analysis to determine independent risk factors for procedure-related complications. *p* < 0.05 was considered significant.

## Results

### Patient and aneurysm characteristics

A total of 30 ruptured wide-necked intracranial aneurysms in 30 patients (12 women, 18 men) were included for analysis. The mean patient age was 55.37 ± 12.04 years (range, 23–75). The Hunt and Hess’s grade was Grade 0 in 6 cases, Grade I in 8 cases, Grade II in 10 cases, Grade III in 5 cases, and Grade IV in 1 case. The location of all the 30 aneurysms was in anterior communicating artery, of which 25 aneurysms were regular in shape and 5 were irregular. Patient and aneurysm characteristics are shown in Table 1.

**Table 1:** Baseline demographics and aneurysm characteristics.

Characteristics	Value
Patients	30
Age (years)	55.37 ± 12.04
Men	18 (60)
Drinking	7 (23.3)
Smoking	9 (30)
Hypertension	14 (46.7)
Diabetes	0 (0)
Hunt–Hess classification	30
0	6 (20)
1	8 (26.7)
2	10 (33.3)
3	5 (16.7)
4	1 (3.3)
Aneurysm shape	30
Irregular	5 (16.7)
Regular	25 (83.3)
Neuroform Atlas stent	30
Double microcatheters technique	23 (76.7)
Single microcatheter technique	7 (23.3)

Values are presented as mean ± standard deviation or number (%).

### Postprocedural angiographic and clinical outcomes

**Figure 1:**
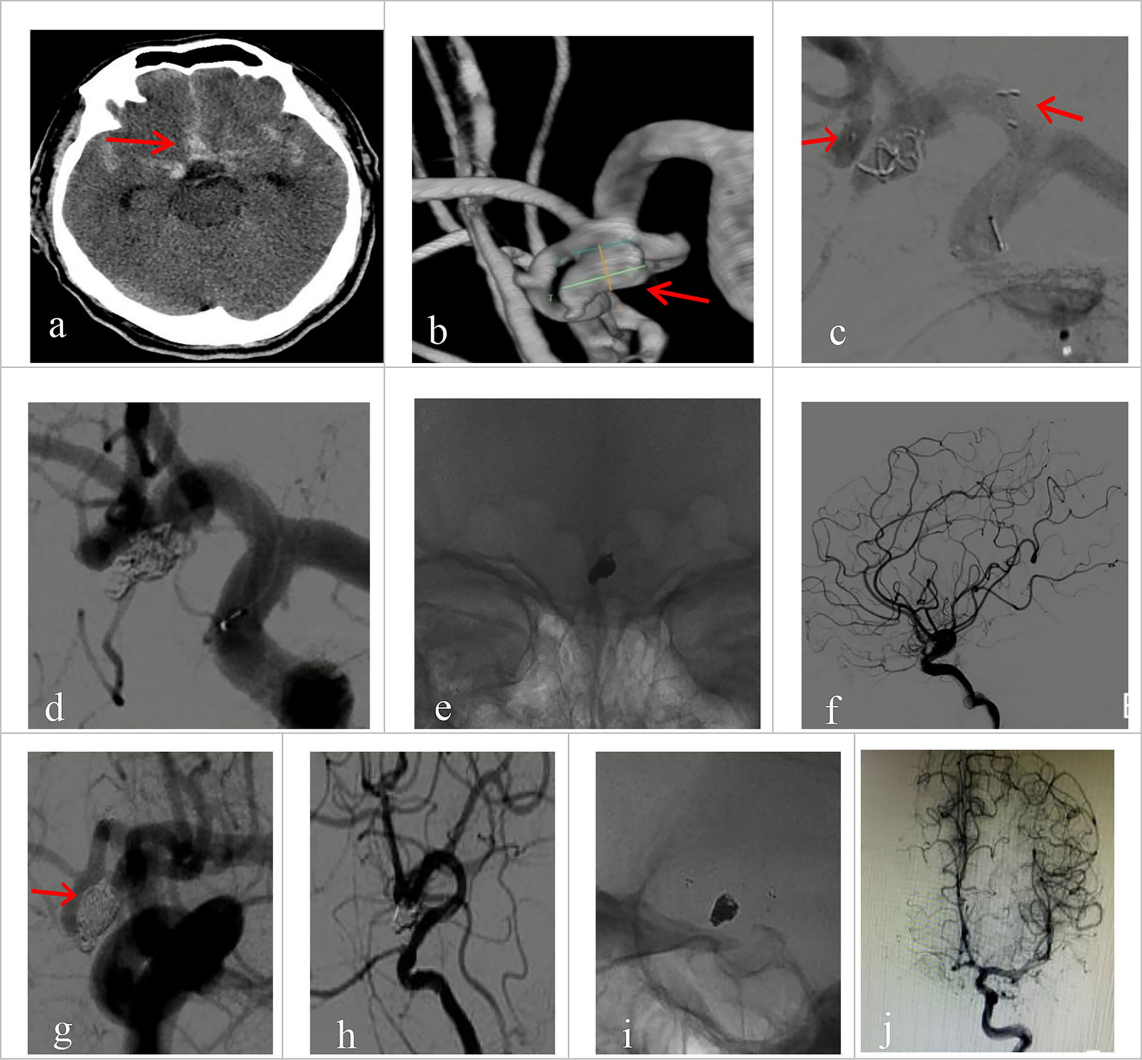
Imaging data of patients with ruptured wide-necked anterior communicating artery (ACoA) aneurysm treated with Neuroform Atlas assisted coiling (dual catheter system). **(a)** Preprocedural head CT showed spontaneous subarachnoid hemorrhage (red arrowhead). **(b)** Preprocedural DSA angiography with 3D reconstruction showed the location of a wide-necked ACoA aneurysm (red arrowhead). **(c)** The stent was positioned as planned and was accurately deployed during the procedure. The left red arrowhead indicates the proximal end of the stent, and the right arrowhead indicates the distal end. **(d)** The coil was delivered after the stent was deployed. **(e)** The stent maintained its expansion after coiling. **(f)** Immediate post-operative angiography (lateral view) demonstrated good patency of the aneurysm-bearing artery and its branch vessels without deficits. **(g–j)** Immediate angiography control showed that the aneurysm was completely embolized (Raymond Grade I), with sound patency of the stent, free of thrombosis. Vessels at all grades had good filling. **(g)** No coil compression 6 month after the procedure was seen on angiography at working projection (red arrowhead indicates the neck of the aneurysm). **(h)** Satisfactory embolization of the aneurysm with no recurrence, no contrast agent stagnation, no thrombosis in the stent. **(i)** Good expansion of the stent on 3D rotating view, without coil compression. **(j)** Parent vessel and its branches had normal fill.

**Figure 2:**
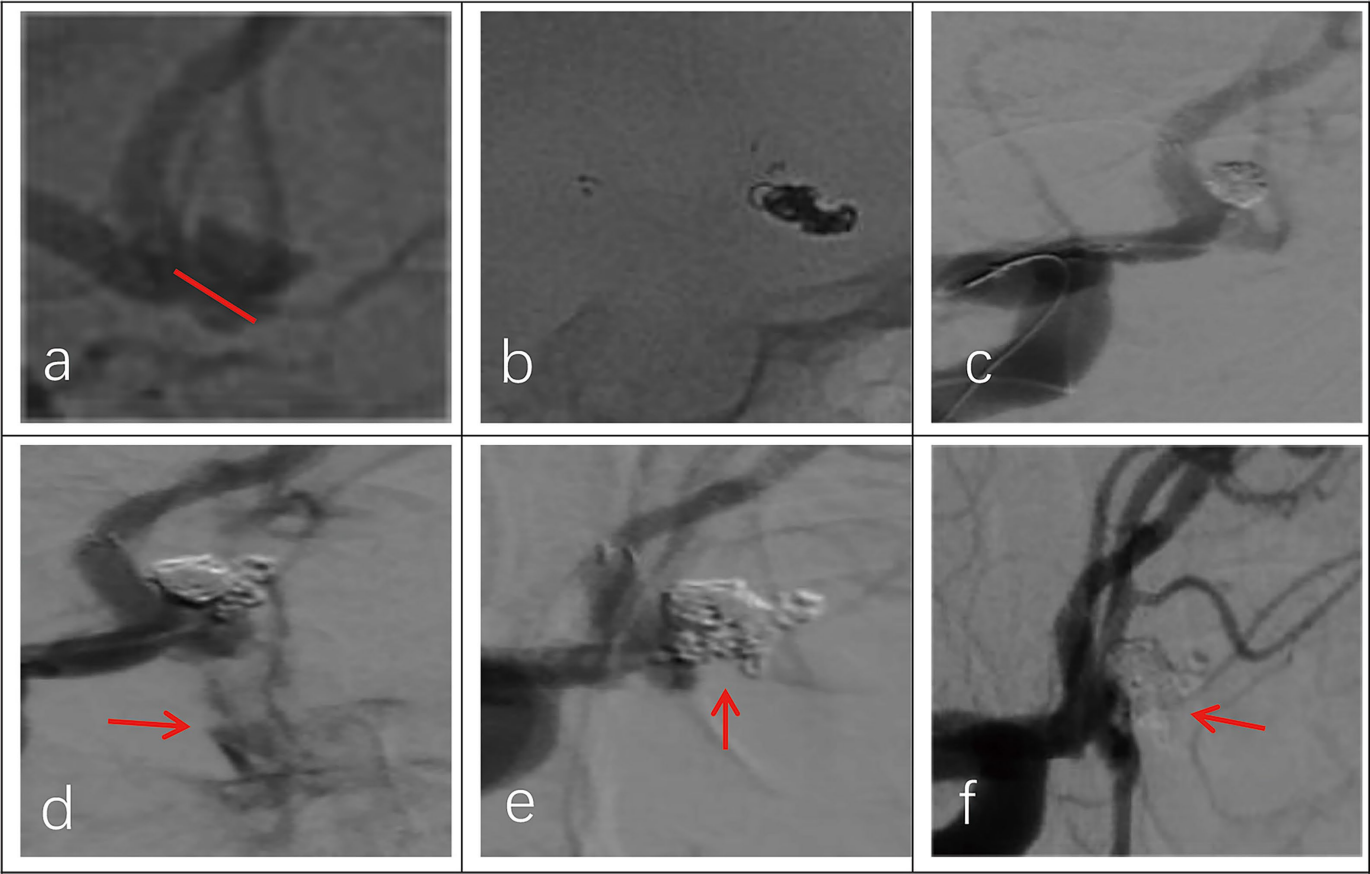
Imaging data of a 44-year-old female patient with a ruptured wide-necked anterior communicating artery (ACoA) aneurysm. **(a)** Location of a ruptured wide-necked ACoA aneurysm (red line). **(b)** The stent was successfully deployed from the ipsilateral A2 to the A1 segment. **(c)** The microcatheter was delivered into the aneurysm sac to place the coils. **(d)** The shape of coil embolization was different from 3D reconstruction of the aneurysm. The aneurysm was packed with more coils until aneurysm partial occlusion (modified Raymond-Roy Class II). Contrast medium leakage from the aneurysm sac (red arrowhead) suggests aneurysm rupture and hemorrhage. **(e)** Following continued rapid coil packing, subsequent angiography revealed no contrast medium leakage from the aneurysm (red arrowhead). **(f)** Follow-up angiography at 6 months showed complete occlusion (modified Raymond-Roy Class I) of the aneurysm (red arrowhead), with no visualization of the aneurysm sac or neck.

**Figure 3:**
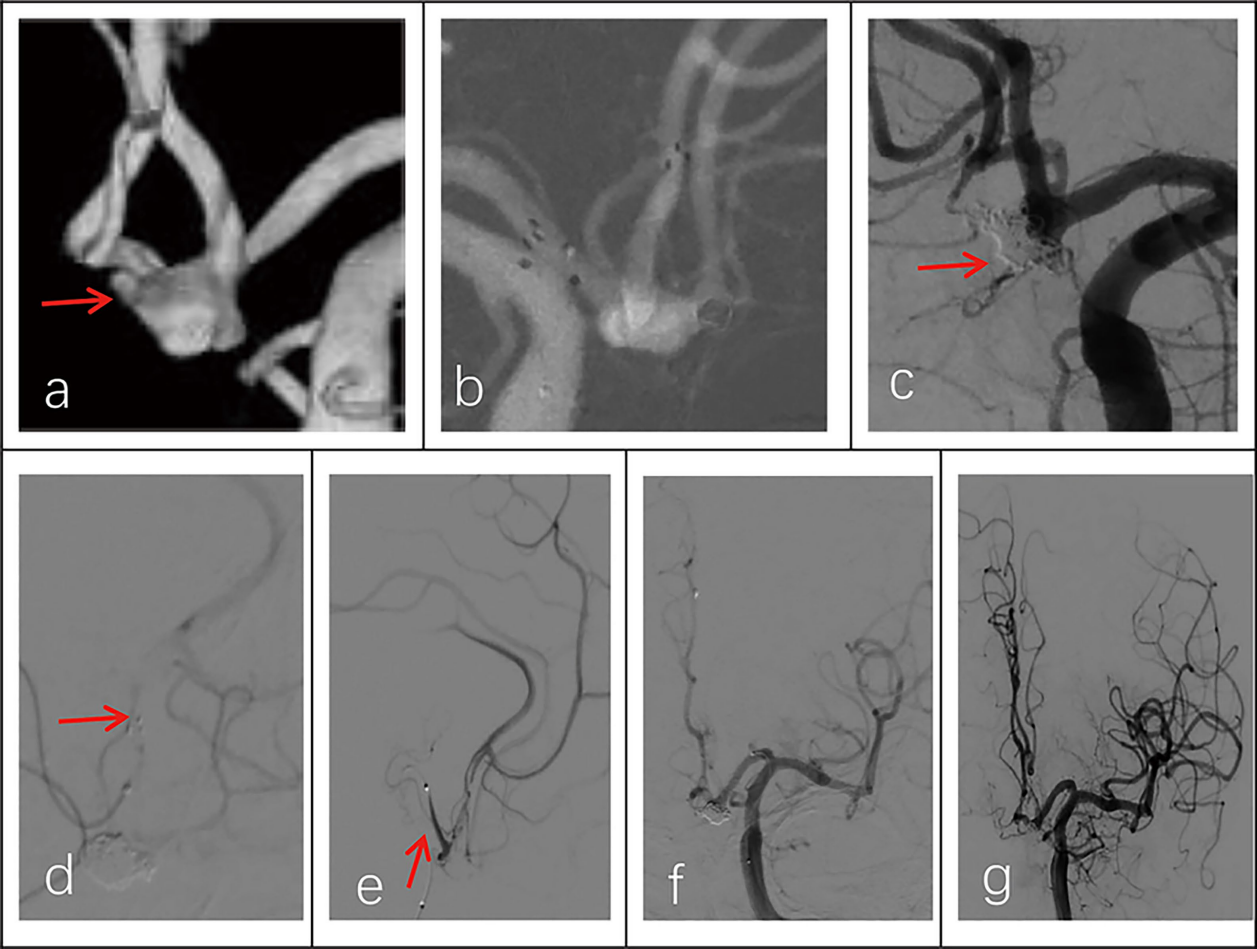
Imaging data of a 58-year-old male patient with a ruptured wide-necked anterior communicating artery (ACoA) aneurysm. **(a)** DSA angiography with 3D showed the location of a ruptured wide-neck ACoA aneurysm (red arrowhead). **(b)** The stent was successfully deployed from the ipsilateral A2 to the A1 segment. **(c)** The microcatheter was inserted into the aneurysm sac through the stent mesh to place the coils. Complete occlusion was achieved under final view (modified Raymond-Roy Class I) (red arrowhead). **(d)** Five hours postembolization developed right hemiparesis. Left ICA angiography showed cross flow to the M1 segment but with a filling defect in the right MCA area. There was thrombosis at the distal end of the stent (red arrowhead) and delayed development of the A2 segment of the left anterior cerebral artery with partial absence of distal branches and terminal vessels. **(e,f)** The microcatheter was delivered into in-stent thrombosis to give an infusion of tirofiban (0.2 mL/kg) (red arrowhead). **(g)** DSA showed that the A2 segment of the left anterior cerebral artery was completely reperfusion, and thrombus was greatly reduced.

All 30 patients had effective Neuroform Atlas stent deployments, with good expansion and location. Twenty-three cases (76.7%) employed the double micro catheter approach, whereas 7 cases (23.3%) employed the single micro catheter technique. Immediate postprocedural angiography showed complete occlusion (RR Grade I) in 18 patients (94.7%) (Figure 1 shows the representative case), neck remnant (RR Grade II) in 0 (0%), and partial occlusion (RR Grade III) in 1 (5.3%). The data of the other 11 patients was unavailable because they refused to accept postprocedural angiography whose follow-up outcomes were good (mRS score ≤2). Clinical outcome was favorable in 29 patients (96.7%) and poor in 1 (3.3%). The mRS score was 3 in the single patient with poor clinical outcomes. Postprocedural angiographic and clinical outcomes are shown in Table 2 and Supplementary Figure 1. Procedure-related complications occurred in three patients (10%), including two cases of hemorrhagic complication and one case of thrombotic complication. No procedure-related death occurred. Contrast medium leakage was discovered during coil delivery in two patients with hemorrhagic complications, but it vanished during the angiography examination following rapid coil delivery. In terms of neurological recovery, mRS score was ≤2 (Figure 2 shows the representative case). One case was found post-procedural acute intrastent thrombosis. With slow injection of tirofiban hydrochloride (0.2 mL/kg) through the guiding catheter, angiography showed disappearance of thrombosis with effective reperfusion and neurological recovery, that was, mRS score ≤2. Postprocedural CT scan showed all patients had satisfactory embolization before smooth discharge from the hospital (Figure 3).

**Table 2:** Postprocedural angiographicand and clinical outcomes.

Characteristics	Value
Neuroform Atlas stent	30
Successful stent placement	30 (100)
Failed stent placement	0 (0)
Initial angiographic results	19
Complete occlusion (Raymond Grade I)	18 (94.7)
Residual neck (Raymond Grade II)	0 (0)
Residual aneurysm (Raymond Grade III)	1 (5.3)
Procedure-related complications	3
Thrombotic complications	1 (3.3)
Hemorrhagic complications	2 (6.7)
mRS score	30
0	22 (73.3)
1	6 (20)
2	1 (3.3)
3	1 (3.3)

Values are presented as mean ± standard deviation or number (%).

### Risk factors for procedure-related complications

Multivariate regression analyses and univariate analyses revealed that none of the variables under investigation had a significant correlation with procedure-related problems (Table 3).

**Table 3:** Potential risk factors for procedure-related complications.

Variable	Procedure-related complications		Unadjusted^a^	Adjusted^b^
	Yes (*n* = 3)	No (*n* = 27)	*p*-value	*p*-value
Age (years)	44 ± 6.00	56.63 ± 11.93	0.085	0.227
Sex			0.054	0.997
Men (*n* = 18)	0 (0)	18 (66.7)		
Women (*n* = 12)	3 (100)	9 (33.3)		
Drinking, yes	0 (0)	7 (25.9)	1.0	
Smoking, yes	0 (0)	9 (33.3)	0.534	
Hypertension, yes	1 (33.3)	13 (48.1)	1.0	
Diabetes, yes	0 (0)	0 (0)	No	
Hunt–Hess classification			0.954	
0 (*n* = 6)	0 (0)	6 (22.2)		
1 (*n* = 8)	1 (33.3)	7 (25.9)		
2 (*n* = 10)	2 (66.7)	8 (29.6)		
3 (*n* = 5)	0 (0)	5 (18.5)		
4 (*n* = 1)	0 (0)	1 (3.7)		
Aneurysm shape			0.064	0.270
Irregular (*n* = 5)	2 (66.7)	3 (11.1)		
Regular (*n* = 25)	1 (33.3)	24 (88.9)		

Values are presented as mean ± standard deviation or number (%) unless otherwise indicated. OR, odds ratio; CI, confidence interval.

a Unadjusted: *p*-values were calculated using the Student’s *t*-test, Wilcoxon rank-sum test and Fisher exact tests.

b Adjusted: Three variables, age, sex and aneurysm morphology, were entered into the multivariate logistic model to calculate the adjusted *p*-value.

## Discussion

Ruptured anterior communicating artery is a common site of intracranial aneurysms, and most of the aneurysms occurring here are wide-necked ones. Coil basketing becomes more challenging when treating wide-necked aneurysms with basic coiling because it may jeopardize the aneurysm cavity’s support. Furthermore, coils are susceptible to herniation or displacement into the parent artery (17). The recurrence rate of aneurysms with wide necks can be considerably decreased using SAC (18, 19). A new self-expandable, semi-open and semi-close cell laser-cut microstent made of nickel-titanium metal alloy (nitinol) is called the Neuroform Atlas stent. It can enhance the embolization of aneurysms and be administered through the tortuous distal tiny channel. This stent was used to treat 113 patients with wide-necked aneurysms in another trial (10). The findings indicated that the rate of aneurysm embolization at the 12-month follow-up was 82 and 88%, respectively. The study’s total complication rate was 6.2%. Intraoperative aneurysm rupture, thromboembolism, coil migration, and stent displacement are the main complications of endovascular embolization for cerebral aneurysms (11, 20–22). While multivariate analysis revealed no independent risk variables, our univariate study suggested that age, sex, and aneurysm shape could be linked to surgical difficulties. Kwon et al. (23) examined the variables linked to surgical problems in Neuroform Atlas stenting for wide-neck cerebral aneurysms and discovered that gender and age were not independent risk factors, which is consistent with our findings. Commercially accessible intracranial stents have been developed since the first Neuroform stent (Stryker Neurovascular) was introduced in 2020. Because of its design, which enables a lower profile delivery, better trackability, and higher conformability to the arterial wall, the Atlas stent may be linked to a higher rate of total occlusion than other stents. The available diameters and lengths are 3.0 to 4.5 mm and 15 to 30 mm, respectively (24). The Atlas stent is linked to a reduced rate of in-stent stenosis and a greater rate of occlusion when compared to the LVIS Jr stent (MicroVention, Inc., Aliso Viejo, CA, United States), according to the Gross’s research (25). Procedure related complications were lower in the Neuroform stent than in the LVIS stents (26). The aforementioned studies have validated the stent’s efficacy and safety in treating ACoA wide-necked aneurysms. Furthermore, as compared to traditional stents, Neuroform Atlas stents offer the following benefits. Wide-neck cerebral aneurysms are treated endovascularly using alternative stents. For wide-neck cerebral aneurysms, a self-expanding open-cell stent device with an antithrombogenic hydrophilic polymer covering treatment produced positive outcomes (27). In 15 individuals with unruptured wide-neck bifurcation aneurysms, a hydrophilic polymer covering was applied (28). In a brief period of time, these investigations proved the effectiveness and safety of stents. It can be administered using a tiny, very compatible microcatheter. The SL-10 or XT-17 microcatheter can be used to distribute Neuroform Atlas of any size to distant vessels. With a diameter of 3.0–4.5 mm, the stent is designed to be inserted into a blood artery that is 2.0–4.5 mm in diameter. Its ability to effortlessly navigate the tortuous parent artery and reach the distal parent vessel is an advantage. Thus, in distal tiny arteries, this stent offers the solution to SAC. As a result, it increases the list of endovascular therapy indications. Furthermore, according to the author’s experience, the stent can grow enough in arteries that have a diameter of at least 1.0 mm.

Stents often have poor wall apposition and fit in narrow, convoluted arteries. Poor wall apposition is the result of close-cell stents like Enterprise and Solitaire being readily distorted at the vessel’s curvature (29). The Neuroform Atlas stent has a smart cell architecture with 8 to 12 alternating struts and a large number of connections. This configuration enhances the stability of the stent and coils in the aneurysm in addition to making it simpler for the coiling microcatheter to pass through the stent cells. It enhances the stent’s compliance and accessibility. Neuroform Atlas adheres to the tortuous section and tracks the vessel’s path. It can be supplied easily and deployed successfully in tortuous blood arteries of comparable size despite the lack of tip design. The Neuroform Atlas stent may also lessen the chance of distal vascular damage.

The open cell of the Neuroform Atlas stent can sustain expansion and precise location in contrast to braided stents. Stable support may be offered by the proximal closed cell structure. The stent has a very low shortening rate and a very high degree of precision after deployment. The high deployment success rate of 93 to 100% may be attributed to this feature, which may prevent endothelial damage from repetitive adjustments (30). Wide-neck aneurysms can be safely and successfully treated with stent-assisted coiling (10, 19, 31–33). A retrospective analysis of 123 patients with unruptured wide-neck aneurysms treated with NeuroForm Atlas stent-assisted coiling revealed that 79.2% of the aneurysms had complete occlusion (23), while another study performed Neuroform Atlas stent-assisted coil embolization on 62 patients with unruptured intracranial wide-neck aneurysms (34). These trials showed that the Neuroform Atlas stent was safe and effective, with a high aneurysm occlusion rate, minimal incidence of perioperative complications, and a high technical success rate. The Neuroform Atlas stent, which is distinguished by low complication rates and limited metal coverage, has comparatively reduced risks of thrombosis and bleeding events when compared to other stent types (such as braided stents). Antiplatelet medication does not raise the risk of thrombotic events in the event of rupture if it is not administered prior to surgery. Thrombosis can be avoided after stent placement by progressively administering 0.2 mL/kg of tirofiban hydrochloride via catheter.

Vasospasm of the anterior cerebral artery may occur in patients who have subarachnoid hemorrhage due to the rupture of an anterior cerebral artery aneurysm. Operating through two microcatheters is extremely challenging due to the vessels’ tiny size. The Neuroform Atlas’s open-cell design allows for the delivery of the stent by a single microcatheter, and the aneurysm sac may be readily accessed by the same catheter with the use of the cross-cell approach. The Neuroform Atlas’s excellent radial force may also lessen parent vascular spasm, and its designs and operations guarantee the safety and seamless development of the surgery. Numerous factors, including inadequate antiplatelet therapy, prolonged surgery duration, intravascular embolic materials linked to thrombogenesis, and poor stent-arterial wall positioning, have been linked to an increased risk of intraoperative thrombosis with the Atlas stent (23, 35, 36). In our investigation, tirofiban was used to treat one patient (3.3%) who had in-stent thrombosis. This resulted in vascular recanalization and positive follow-up outcomes (mRS score <2). It should be mentioned as well that an open stent does not provide adequate neck protection for aneurysms with broad necks. Based on our clinical experience, selecting a Neuroform Atlas stent with a greater diameter can safeguard the aneurysm further without raising the risk of in-stent thrombosis.

Due to a number of limitations, such as the retrospective data collection and the small number of patients with ruptured aneurysms recruited, it is unable to completely rule out the impact of potential selection bias. Moreover, there was no actual control group to assess the device’s safety and effectiveness. Furthermore, prolonged follow-up is required to evaluate the durability of appropriate obliteration because the follow-up time was brief (less than 2 years). Therefore, more clinical trials should be conducted to compare the Neuroform Atlas with other devices and conduct laboratory studies to improve its technological flaws.

## Conclusion

The Neuroform Atlas stent has a low risk of complications and is a successful treatment for ruptured wide-necked ACoA aneurysms. To verify the long-term safety and effectiveness, prospective multicenter trials with a bigger sample size and long-term follow-up are required.

## Data Availability

The original contributions presented in the study are included in the article/Supplementary material, further inquiries can be directed to the corresponding author.
