# Supplementary figures and images for: Neuroform Atlas stent-assisted coiling of ruptured wide-necked anterior communicating artery aneurysms
Source: Front Neurol. 2025 Oct 8;16:1674012. doi: 10.3389/fneur.2025.1674012 (PMC12541818; doi:10.3389/fneur.2025.1674012)

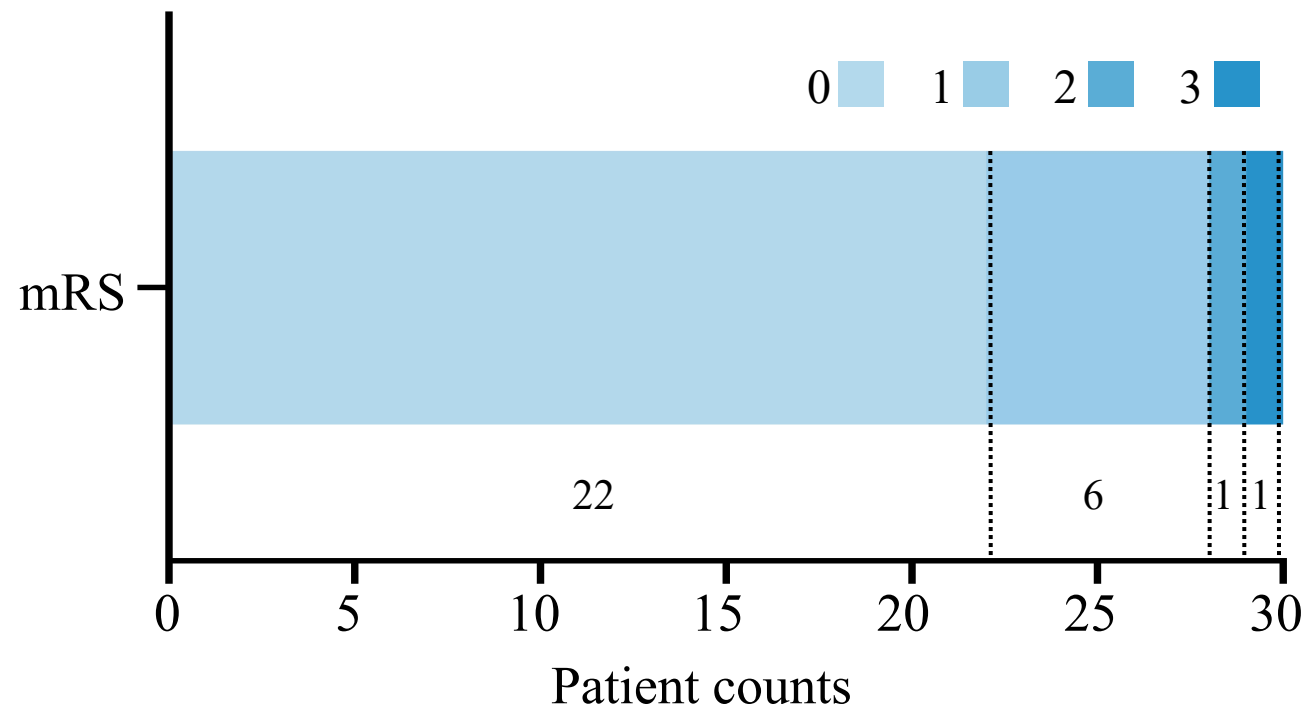

Supplement: Supplementary file 1 [file Data_Sheet_1.PDF]
